# A Unique Case of Candida auris Infection Presenting With Hydropneumothorax and Bronchopleural Fistula: A Diagnostic and Therapeutic Challenge

**DOI:** 10.7759/cureus.73147

**Published:** 2024-11-06

**Authors:** Bodhisatwa Choudhuri, Madhuparna Chakraborty, Pratik Biswas

**Affiliations:** 1 Critical Care and Rheumatology, Parkview Super Speciality Hospital, Kolkata, IND; 2 Critical Care, Parkview Super Speciality Hospital, Kolkata, IND; 3 Pulmonology, ILS Hospital, Howrah, IND

**Keywords:** antifungal resistance, bronchopleural fistula, candida auris, critical care infection, fungal pleural effusion, hydropneumothorax, icu-acquired infection, invasive candidiasis, multidrug-resistant fungal infection, posaconazole

## Abstract

*Candida auris* is an emerging multidrug-resistant fungal pathogen that has become a significant global health concern, particularly in critically ill patients within hospital settings. It is known for its high mortality rates, diagnostic challenges, and frequent misidentification, which delays appropriate treatment. We present a case of a 72-year-old male with diabetes and hypertension who initially presented with a persistent cough, hemoptysis, and fever and was initially suspected of having pulmonary tuberculosis. Despite tests negative for tuberculosis, empirical anti-tubercular treatment and antibiotics were initiated. However, subsequently, the patient deteriorated, developing hydropneumothorax and bronchopleural fistula, suggesting a different diagnosis. Advanced fungal cultures from endotracheal secretions later confirmed *Candida auris* infection. Given the concern for antifungal resistance, initial treatment with caspofungin was switched to posaconazole, leading to marked clinical improvement. After 21 days of hospitalization, the patient was discharged and continued posaconazole for two months, with full recovery by the three-month follow-up. This case represents the first reported instance of *Candida auris* infection complicated by hydropneumothorax and bronchopleural fistula, a rare and severe pulmonary manifestation. It underscores the diagnostic difficulties associated with *Candida auris*, which often mimics other infections like tuberculosis, and highlights the importance of advanced diagnostic techniques. The case also emphasizes the utility of posaconazole in managing resistant *Candida auris* infections and the need for heightened clinical suspicion of this pathogen in critically ill patients who do not respond to conventional therapies.

## Introduction

Candida auris is an emerging multidrug-resistant fungal pathogen [1], first isolated in 2009 in Japan. Since then, it has been identified in several countries, including India [2], and is now recognized as a serious global health threat. Candida auris is predominantly found in hospital settings, particularly in intensive care units (ICUs), affecting immunocompromised patients and those with prolonged hospital stays. This pathogen is known for its ability to cause secondary infections, such as sepsis, and has a high mortality rate, particularly in critically ill patients [1].

The prevalence of Candida auris in India has been rising, with several outbreaks reported in hospitals across the country. In Indian ICUs, Candida auris has been identified as a leading cause of candidemia, especially in patients receiving prolonged ventilatory support [2]. Globally, Candida auris is recognized for its ability to persist on healthcare surfaces for extended periods, leading to rapid transmission within hospital settings [3].

Candida auris infections often present with non-specific symptoms like fever, chills , or signs of sepsis, especially in critically ill patients with prolonged hospital stays. Diagnosis typically involves culture, MALDI-TOF mass spectrometry, or PCR methods, with antifungal susceptibility testing guiding treatment. It is challenging to treat because it often exhibits multi-drug resistance to common antifungal agents, including azoles, echinocandins, and amphotericin B, limiting effective treatment options. This report presents the first documented case of Candida auris infection complicated by hydropneumothorax and bronchopleural fistula. This rare clinical presentation highlights the challenges of diagnosing and managing Candida auris infections, particularly in patients with atypical presentations.

## Case presentation

A 72-year-old male, known diabetic and hypertensive, presented with a two-month history of persistent cough, low-grade fever, and occasional hemoptysis. His initial chest X-ray was unremarkable, but a CT scan revealed cavitary lesions in the upper lobe of the right lung. All necessary investigations were done to determine the cause of the cavitary lesion, but they were either negative or inconclusive. Despite negative Mycobacterium tuberculosis PCR and gene expert testing, the patient was empirically started on anti-tubercular therapy (ATT) (rifaximin 600 mg, isoniazid 300 mg, ethambutol 1100 mg, pyrazinamide 1600 mg - once daily) and broad-spectrum antibiotics (faropenem 200 mg thrice daily for five days) due to clinical suspicion of pulmonary tuberculosis (TB). However, he was non-compliant with the treatment regimen and stopped ATT after 10-12 days due to gastritis and nausea.

**Figure 1:**
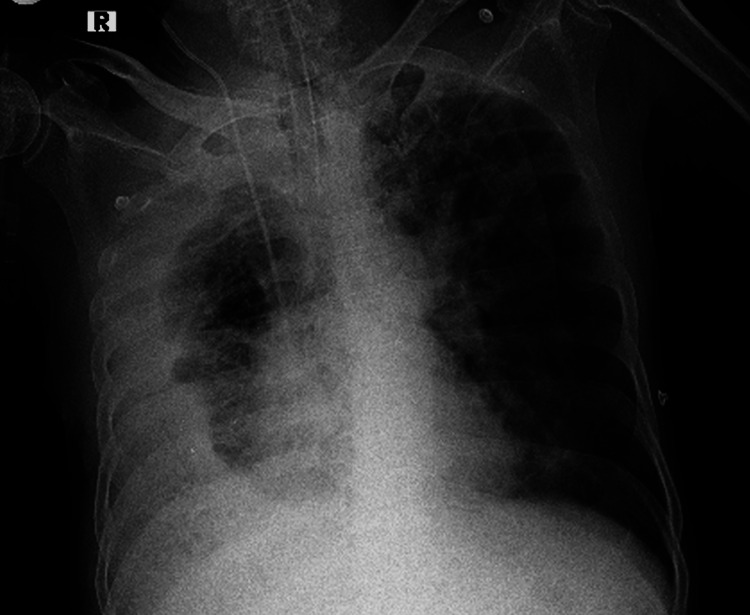
Chest X-ray AP view showing a dependent opacity with lateral upward sloping of a meniscus-shaped contour on the right side suggestive of right-sided pleural effusion. It also shows a central venous catheter at the right internal jugular vein and endotracheal tube in situ. AP view: Anterior-posterior view

The patient developed respiratory distress and was admitted to a local hospital, where he was immediately intubated and ventilated due to severe respiratory acidosis. Chest X-ray showed a right-sided massive pleural effusion (Figure 1).

An intercostal drain (ICD) was placed, but cultures and pleural fluid analyses, including TB PCR, remained inconclusive. Empirical therapy with broad-spectrum antibiotics (IV meropenem 1 gm thrice daily & IV doxycycline 100 mg twice daily) and caspofungin (IV 70 mg loading, followed by 50 mg once daily) was initiated, and anti-tubercular medications were discontinued. The patient's condition initially improved, with resolution of fever, stabilization of symptoms for 48 hours, and subsequent extubation.

**Figure 2:**
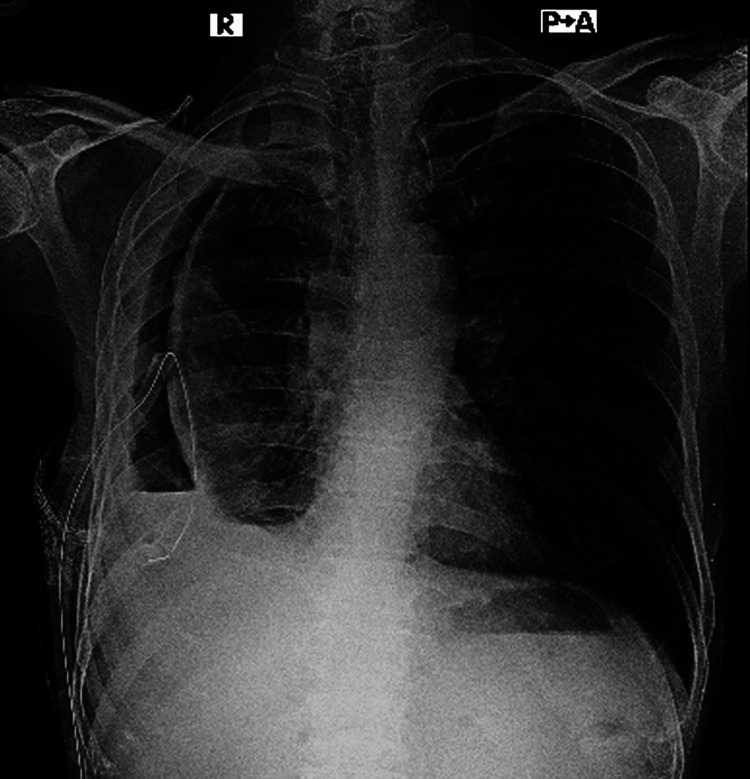
Chest X-ray PA view shows right-sided horizontal air−fluid interface suggestive of hydropneumothorax along with rim of pleural thickening with fibrosis and intercostal chest tube in situ. PA view: posterior-anterior view

**Figure 3:**
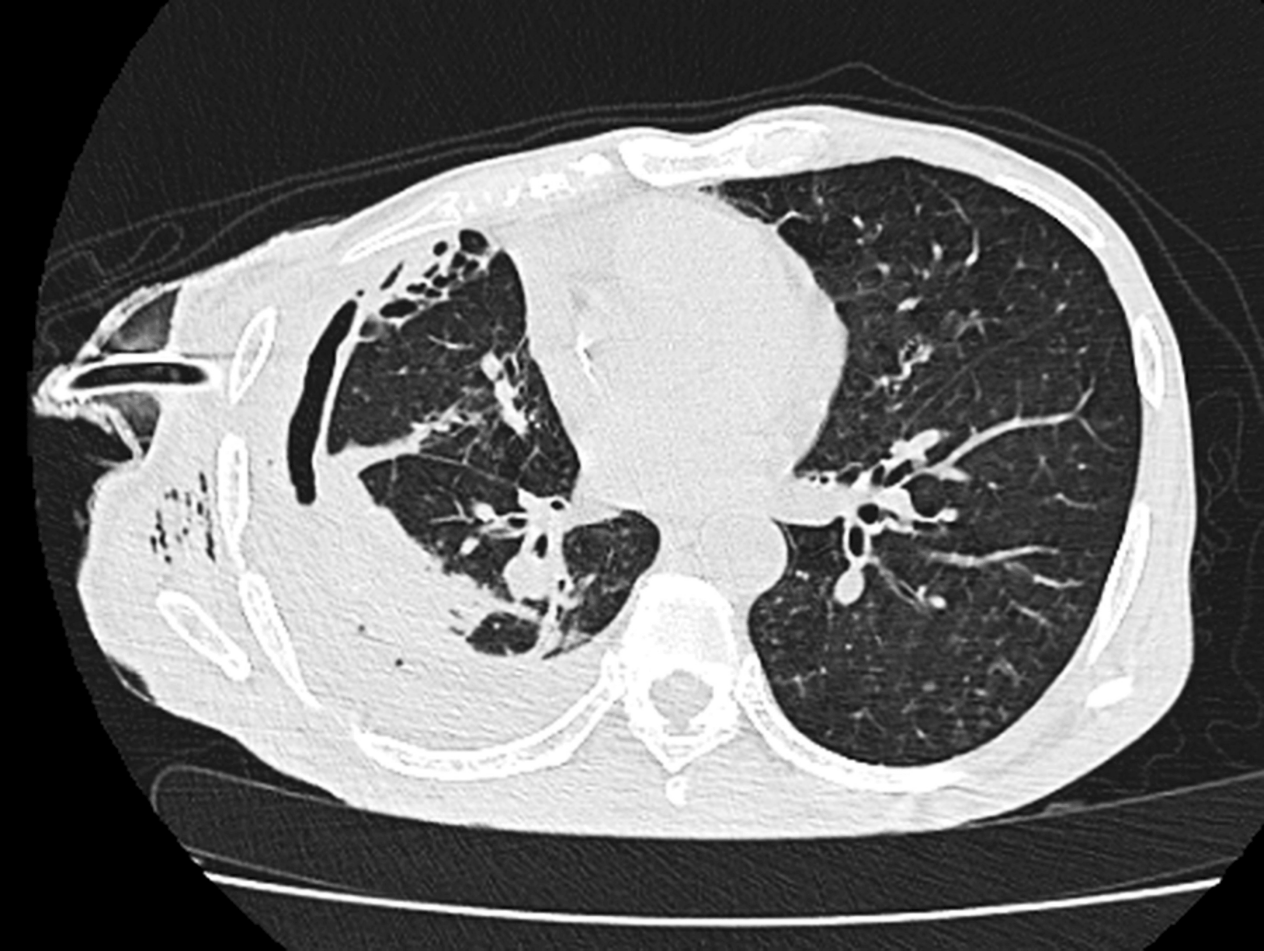
A CT Thorax cut shows consolidation in the right lung, hydropneumothorax with pleural thickening, and volume loss of the right lung. The intercostal chest tube is in situ, and minimal surgical emphysema is present in the subcutaneous plane.

However, his condition soon deteriorated again, with recurrent pneumothorax and the development of a bronchopleural fistula. He required high oxygen support and bilevel positive airway pressure (BiPAP) assistance as respiratory distress worsened. A repeat chest X-ray demonstrated persistent hydropneumothorax and lung collapse (Figure 2), while a contrast-enhanced CT (CECT) thorax confirmed fibrosis of the lung margin (Figure 3). The patient was referred to our hospital for further management.

Upon arrival, the patient was in critical condition, with a Glasgow Coma Scale (GCS) score of 3/15, severe respiratory acidosis (pH 7.28, PaCO_2_ 78 mmHg, PaO_2_ 59 mmHg, lactate 4.3mmol/L), and hemodynamic instability requiring inotropic support. He was immediately intubated and ventilated again. Clinical and radiological signs continued to suggest tuberculosis, but all investigations, including PCR from bronchoalveolar lavage fluid (BALF) and endotracheal (ET) secretion samples, remained negative for TB.

Empirical broad-spectrum antibiotic therapy, including ceftazidime-avibactam (IV 2.5gm thrice daily), aztreonam (IV 2gm thrice daily), and oseltamivir (oral 75mg twice daily) were initiated, alongside caspofungin (IV 50mg once daily) for antifungal coverage. Despite sterile blood and urine cultures, bronchoalveolar lavage fluid (BALF) and endotracheal (ET) secretion samples revealed budding yeast. Both BALF and ET secretion sample cultures confirmed the presence of Candida auris. Caspofungin was empirically switched to posaconazole (IV 300mg twice daily on the first day, then from the second day onwards 300mg once daily) due to suspected antifungal resistance, and antibacterial coverage was continued for possible secondary bacterial infection. Subsequently, the fungal antibiotics sensitivity report showed growth of Candida auris, which was sensitive to posaconazole, voriconazole, and isavuconazole but resistant to echinocandins.

The patient's clinical condition gradually improved with the normalization of inflammatory markers (CRP, procalcitonin, and total counts) (Table 1) and successful extubation after seven days. Rehabilitation with physiotherapy and speech therapy was initiated. Despite the critical illness, the patient did not develop any multi-organ dysfunction.

**Table 1:** Laboratory investigations: Baseline – on the day of admission to our hospital (Day 0), on the day of extubation (Day 7), and on the day discharge (Day 21) CRP: C-reactive protein, WBC: White blood corpuscles, AST: Aspartate aminotransferase, ALT: Alanine aminotransferase, NT-proBNP: N-terminal pro B-type natriuretic peptide

Tests	Results – On admission	Results – On the day of extubation	Results – On the day of discharge	Reference ranges
Haemoglobin	9.2 g/dl	10.7 g/dl	11.2 g/dl	14-18 g/dl
WBC Count	11040 cells/mm^3^	10600 cells/mm^3^	9760 cells/mm^3^	4500-11000 cells/mm^3^
Platelets	165000 cells/mm^3^	180000 cells/mm^3^	230000 cells/mm^3^	150000-350000 cells/mm^3^
CRP	130 mg/l	43.2 mg/l	8.4 mg/l	<3 mg/l
Urea	69.47 mg/dl	61.52 mg/dl	37.8 mg/dl	6-24 mg/dl
Creatinine	1.02 mg/dl	1.28 mg/dl	1.06 mg/dl	0.6-1.2 mg/dl
Sodium	153 mEq/l	136 mEq/l	141 mEq/l	136-142 mEq/l
Potassium	4.1 mEq/l	4.3 mEq/l	3.9 mEq/l	3.5-5 mEq/l
Calcium	7.18 mg/dl	7.74 mg/dl	8.32 mg/dl	8.2-10.2 mg/dl
Magnesium	1.45 mEq/l	2.18 mEq/l	1.86 mEq/l	1.4-2.1 mEq/l
Total Bilirubin	1.7 mg/dl	1.3 mg/dl	0.6 mg/dl	0.1-0.3 mg/dl
Albumin	2.15 g/dl	2.84 g/dl	3.26 g/dl	3.5-5 g/dl
AST	64 U/l	74 U/l	56 U/l	10-40 U/l
ALT	67 U/l	56 U/l	48 U/l	10-55 U/l
Procalcitonin	3.08 ng/ml	0.8 ng/ml	<0.05 ng/ml	<0.05 ng/ml
Troponin-I	<0.04 ng/ml	-	-	<0.04 ng/ml
NT-ProBNP	286 pg/ml	-	-	<125 pg/ml
Beta D-Glucan	134 pg/ml	-	-	<60 pg/ml
Urine Pus Cells	4-6/hpf	-	-	0-2/hpf

**Figure 4:**
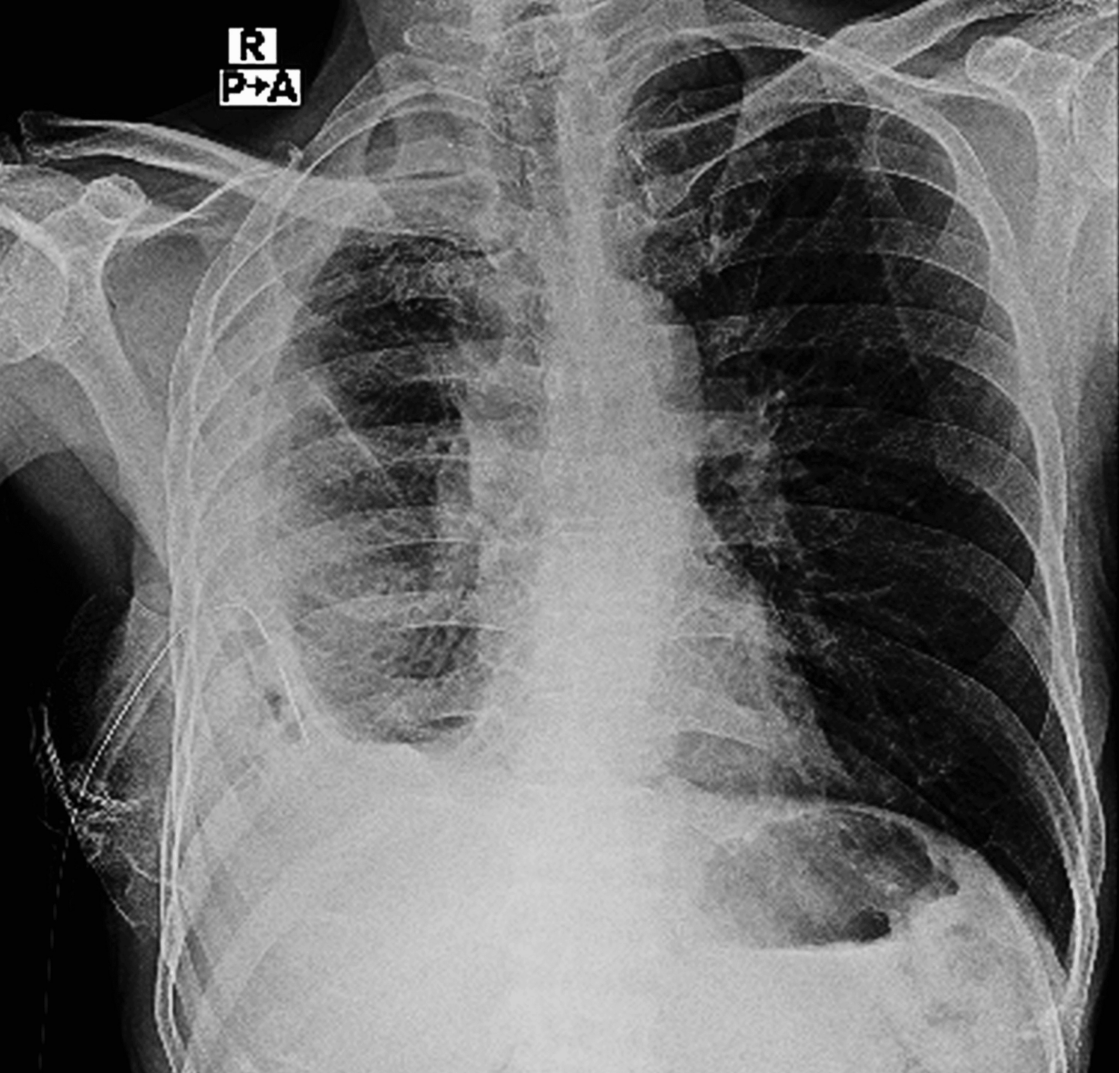
Chest X-ray PA view shows right-sided pleural effusion with resolution of air component of hydropneumothorax, with volume loss of lung on the right side with right intercostal chest drain in situ.

A follow-up chest X-ray revealed persistent hydropneumothorax with right lung collapse and fibrosis. Progressive lung re-expansion was achieved through pressurized suction via ICD (Figure 4). After 21 days of hospitalization, the patient was discharged on oral posaconazole therapy (300mg once daily).

At the one-month follow-up, the patient remained on antifungal therapy. Chest imaging demonstrated residual pneumothorax and lung scarring, but the patient was asymptomatic. Posaconazole therapy was continued for 60 days, and by the three-month follow-up, the patient had resumed normal activities.

## Discussion

Candida auris has been increasingly recognized for its capacity to cause invasive infections in hospital settings [4]. While bloodstream infections and sepsis are the most common presentations, pulmonary involvement, such as lung abscesses or cavitations, is rare but can occur in critically ill patients [5]. In this case, the patient developed hydropneumothorax and bronchopleural fistula, a previously unreported complication of Candida auris infection. Although fungal pleural effusions and empyema caused by Candida species have been reported, the progression to pneumothorax and bronchopleural fistula is extremely uncommon. Fungal elements, such as Candida auris, can invade pleural spaces either through direct extension from adjacent lung lesions or via hematogenous spread. In this case, the formation of a bronchopleural fistula may have resulted from severe infection-induced necrosis of the lung parenchyma, causing communication between the bronchial tree and the pleural space [6]. This case suggests that severe, untreated Candida auris infections can lead to destructive pulmonary pathology, particularly in patients with underlying lung disease or immunosuppression [4].

The diagnostic challenges of Candida auris infection are well-documented. Routine fungal culture methods often misidentify Candida auris as other Candida species like Candida haemulonii, Candida famata, Candida lusitaniae, and Candida parapsilosis, etc., leading to appropriate treatment delays [7]. Diagnosing Candida auris in cases where pulmonary symptoms are predominant can be particularly challenging due to its clinical presentation, which can mimic other infectious diseases, such as pulmonary tuberculosis. This misdiagnosis is compounded by the fact that tuberculosis and fungal infections can co-exist, making the clinical differentiation even more complex [4]. In this case, the delay in identification, due to its atypical presentation and the initial suspicion of tuberculosis, underscores the need for specialized laboratory techniques such as matrix-assisted laser desorption/ionization time-of-flight (MALDI-TOF) to diagnose Candida auris [2,7] accurately. The unavailability of rapid antifungal susceptibility testing methods further complicates management, as most healthcare facilities lack the resources to perform these tests routinely [2].

Managing Candida auris infections remains challenging due to its resistance to multiple antifungal agents. The 2016 Infectious Diseases Society of America (IDSA) guidelines recommend using echinocandins as the first-line therapy for Candida auris infections [8]. However, increasing reports of echinocandin-resistant strains necessitate using alternative agents, such as amphotericin B or newer triazoles like posaconazole and isavuconazole, particularly in resistant cases [2,8]. For the management of multidrug-resistant Candida auris infections, several newer antifungal agents like ibrexafungerp, rezafungin, fosmanogepix, and ravuconazole have shown promise [9]. 

In this case, initial treatment with caspofungin was switched to posaconazole due to concerns about resistance. Posaconazole is a broad-spectrum antifungal with activity against many resistant strains of Candida, including Candida auris. Studies have shown that posaconazole has a good safety profile and is effective in treating resistant infections, especially when combined with other antifungal agents [10,11]. The drug inhibits the synthesis of ergosterol, an essential component of fungal cell membranes, which disrupts the cell membrane's integrity and leads to fungal cell death. Posaconazole's role in managing Candida auris infections is supported by its broad spectrum of activity, including against strains resistant to fluconazole, voriconazole, and echinocandins [8].

Our patient's switch to posaconazole markedly improved clinical symptoms and radiological findings. Continued treatment with posaconazole for 60 days ensured complete resolution of the infection without further complications.

## Conclusions

This case highlights the importance of considering Candida auris in the differential diagnosis of critically ill patients, especially those who do not respond to broad-spectrum antibiotics or antifungal agents and negative tuberculosis PCR tests despite features suggesting the same. Early identification of Candida auris through advanced diagnostic methods is crucial for timely and appropriate treatment. The use of posaconazole in this case underscores its potential role in managing resistant Candida auris infections, particularly in cases complicated by pulmonary involvement, such as hydropneumothorax and bronchopleural fistula. Strict infection control measures remain essential to prevent the spread of Candida auris in healthcare settings. Further research is needed to explore the full clinical spectrum of Candida auris infections and to optimize treatment strategies for resistant cases.
